# Feasibility of implementing an innovative manual handling risk assessment training program for staff working in long-term care

**DOI:** 10.1186/s13690-023-01074-7

**Published:** 2023-04-12

**Authors:** Natasha K Brusco, Christine Graven, Leanne Boyd, Helen Kugler, Helen Dawes, Helen Rawson, Lynne Clayton, Suzanna Tan, Victoria A Goodwin, Abi J Hall, Nicholas F Taylor

**Affiliations:** 1 Rehabilitation, Ageing and Independent Living (RAIL) Research Centre, Monash University, Victoria, Australia; 2 Eastern Health, Box Hill, Australia; 3 School of Allied Health Human Services and Sport, La Trobe University, Victoria, Australia; 4 Clinical Education and Research Institute, Cabrini Health, Malvern, Australia; 5 College of Medicine and Health, University of Exeter, Exeter, UK; 6 Nursing and Midwifery, Monash University, Victoria, Australia

**Keywords:** Manual handling, Risk assessment, Feasibility, Qualitative research, Nurses, Long-term care, Occupational health

## Abstract

**Background:**

The Risk Assessment for moving Individuals SafEly (RAISE) program is a hospital-based manual handling nursing training program. RAISE involves upskilling on continual risk assessment during patient-assisted movements. RAISE aims to optimise staff and patient safety while providing the patient with movement and rehabilitation opportunities. Implementation of RAISE in the hospital setting has been established. The aim of this study was to explore the feasibility of implementing RAISE in the long-term care setting.

**Methods:**

We examined three feasibility domains: acceptability, practicality, and limited efficacy (observed nursing behaviour change which has the potential to reduce nursing injuries), using a prospective pilot pre-post design in the long-term care setting. Staff completed a 4-hour training session on RAISE delivered by two physiotherapists, followed by 8 h of supported behaviour change in the workplace. Staff acceptability and practicality of incorporating risk assessment strategies into manual handling approaches were explored through pre- and post-training staff surveys and a semi-structured interview. Resident acceptability of manual handling practices was explored via survey data collected after the RAISE training. Pre to post-training changes in staff knowledge and behaviour were examined through the pre- and post-training staff survey, and observation of staff assisting resident movement.

**Results:**

Two enrolled nurses and five residents participated. Staff reported the RAISE program was acceptable and practical to implement in the long-term care setting. There were no adverse events or safety concerns. Staff reported the RAISE program provided guidance and enhanced staff empowerment to make decisions during assisted resident movement. There were 26 observed resident-staff manual handling interactions recorded, with 13 pre-training and 13 post-training. Post-training, RAISE skills had improved and were completed 100% of the time, except for completing a physical risk assessment which improved from 46 to 85%, demonstrating limited efficacy. Residents reported it’s important for staff to be trained on how to assist them to mobilise and they found the concept of the RAISE program acceptable.

**Conclusions:**

This pilot study supports the feasibility of long-term care facilities participating in future studies testing the effectiveness and cost-effectiveness of the Risk Assessment for moving Individuals SafEly (RAISE) patient and resident manual handling program.

**Supplementary Information:**

The online version contains supplementary material available at 10.1186/s13690-023-01074-7.

## Background

Assisting patients to move in a hospital, or residents to move in a long-term care setting comes with risk to both the staff and the patient/resident [1–3]. Staff are at risk of musculoskeletal injuries while physically assisting movement [2, 3], and patients are at risk of falling during the movement [4], or conversely becoming deconditioned if they do not participate in movement [4]. This raises interdependent priorities; the need to promote and preserve mobility in patients and residents, while concurrently preventing falls and ensuring staff safety during assisted movement [4, 5]. It is of concern that multiple international systematic reviews have demonstrated that current manual handling training programs have not been able to reduce nursing staff musculoskeletal injuries [6, 7], and that this comes at a great cost [8].

Support for these interdependent priorities are embedded into the recent Royal Commission into Aged Care Quality and Safety in Australia, which has recommended that ‘…care and supports should, as far as possible, emphasise restoration and rehabilitation, with the aim of maintaining or improving older people’s physical and cognitive capabilities, and supporting their self-determination’ (10 p206). To support resident care and staff safety, the Royal Commission also recommended setting minimum staffing levels and minimum qualifications for staff providing care. Over recent years there has been a change in personnel who provide direct care in long-term care facilities in Australia, with reducing numbers of Registered and Enrolled Nurses, and less qualified Personal Care Assistants now accounting for about 70% of the direct care workforce [9].

The Risk Assessment for moving Individuals SafEly (RAISE) is a new manual handling training based on continual risk assessment during patient-assisted movements, to optimise safety aspects of the task being performed. Based on previous work, an expert multidisciplinary working-party (including nurses, allied health clinicians, and work health and safety staff) created the RAISE manual handling training program, using Kolb’s Experiential Learning Theory to inform the process [10, 11]. RAISE focuses on multiple factors including staff training, policy change and addressing organisational factors. A previous RAISE feasibility study was conducted in the acute and sub-acute hospital settings, and yielded positive results, indicating that the RAISE program taught nurses to better identify factors associated with risk to themselves and their patients, and gave them improved skills to assist patients to move [12]. Furthermore, these skills were immediately incorporated safely into clinical practice and maintained at six months post-training. In addition, it was concluded that this risk assessment manual handling training improved confidence and empowered nurses to change their practice and provide safe mobility-related care [11]. To date, RAISE pilot work has not included long-term care facilities, despite the high frequency of nursing and care staff injuries in this setting [13], where staff are exposed to physical, organisational and psychological factors which can contribute to musculoskeletal disorders [14]. In long term care, there is limited evidence of interventions that effectively reduce staff musculoskeletal injuries, with interventions that focus on multiple factors being more successful [14].

This feasibility study aimed to explore staff and resident acceptability, practicality, and limited efficacy of the RAISE training program when implemented in the long-term care setting. It was hypothesised that implementing RAISE would be acceptable to the staff and residents, and that implementing RAISE would be perceived to be practical and safe by staff. It was also hypothesised that staff in a long-term care setting who participated in RAISE training would demonstrate knowledge gain and positive behaviour change when assisting residents to perform mobility tasks in the workplace, aligned to the RAISE training principles.

## Methods

### Ethics approval and reporting guidelines

The study protocol was approved by the Eastern Health Human Research Ethics Committee (Project Number: LR22-022-86171). All participants (staff and residents) provided written informed consent prior to data collection. The design and reporting of this feasibility study was informed by the theoretical framework outlined by Bowen et al., which includes eight domains of feasibility [15]. Of the eight domains, the current study examined three of these domains; acceptability, practicality and limited efficacy [15]. These three domains were selected as they were key to planning then next phase of the overarching program of research. Lancaster and Thabane’s guidelines for reporting non-randomised feasibility studies have informed the reporting of this study [16], with the CONSORT checklist for pilot and feasibility studies [17] attached as Appendix 1. There were no major changes to the methods or outcomes after the study commenced.

### Design, setting and participants

Acceptability was reported from staff and resident perspectives and practicality was reported from the staff perspective. Limited efficacy refers to staff gains in knowledge and positive behaviour change following participation in the RAISE program. We used a prospective pre-post design to evaluate the RAISE program, using the Kirkpatrick Model to provide a system for appraisal [18]. The Kirkpatrick model is based on four levels of assessment [18] and in the context of the current study they included: [1] participant reaction to the training via the focus group, [2] assessment of participant learning via changes in the pre- and post-training surveys, [3] change to clinical practice at the bedside by the participants via an observational audit, and [4] impact on the key outcomes of interest, i.e., staff injuries, noting this fourth level of assessment was out of scope for the current feasibility study. To address the aims, there were several components to this feasibility study. The setting was a 30-bed permanent long-term care facility operating within a large public healthcare network (Eastern Health) in Melbourne, Australia. As this was a feasibility study, sample size was not estimated a priori, this was a sample of convenience to inform design of a larger study.

#### Staff participants

Staff who provided direct care to the residents, involving manual handling tasks, over three consecutive rostered day shifts were eligible to participate in the study. The service manager informed staff about the project during a regular staff meeting and asked the staff for expressions of interest. Following an expression of interest, staff had a 1:1 discussion with a member of the research team to discuss the project and ensure written informed consent was provided prior to commencement. The research team aimed to recruit between 4 and 6 staff participants. At the time of participant recruitment, it was noted by the service manager that since the beginning of the Covid-19 pandemic in early 2020 until current, the service had experienced chronic short staffing.

#### Resident participants

For residents to be eligible, they must also have received care by a RAISE-trained staff member during the study period. While on-site, a member of the research team approached the residents asking for interest to participate. This continued until 5 residents had expressed interest and provided written informed consent to participate. Consent / proxy consent was not sought for potential study participation from residents who were considered by the facility manager to have a level of cognitive impairment or limited English language proficiency that impeded their ability to give informed consent.

### Intervention

The RAISE program was designed to teach healthcare workers continual risk assessment before, during and after assisting people to move, using the pillars of Task, Individual, Load, and Environment (TILE). RAISE focuses on multiple factors including staff training, policy change and addressing organisational factors. Details of the intervention published previously [12, 19]. Training at the long-term care facility involved staff participants attending a 4-hour RAISE training session, incorporating both a theoretical component utilising a program manual (with photographic illustrations outlining bedside risk assessment decision trees) and digital presentation, and a practical component with a competency review. This session was facilitated by two trainers (CG and HK). The trainers were experienced physiotherapists, and they assisted the staff participants to practise new skills via role-playing scenarios to replicate common resident physical and functional presentations encountered in the workplace. An audit of manual handling equipment at the facility was conducted to ensure that the staff had access to required items to support their decision processes for safe manual handling practices.

The 4-hour RAISE training session was followed by an 8-hour supported behaviour change in the workplace (during the morning shift). One of the trainers (CG) attended a morning nursing shift to provide tuition to the staff participants while they performed their manual handling tasks with residents. This on-site support training session enabled staff participants to receive additional demonstrations, practice, feedback and collaborative assistance, to build on the information that had been conveyed during the 4-hour training session. All staff had previously participated in a compulsory standardised task and technique-based manual handling training program conducted by the healthcare network.

### Data collection

#### Staff participants

Staff completed pre- and post-training surveys, to capture staff acceptability, practicality and limited efficacy. The pre- and post-training surveys included closed and open questions, which sought to understand knowledge of incorporating risk assessment into resident manual handling tasks, as well as the practicality and acceptability of integrating risk assessment into manual handling tasks. Surveys were paper-based and were provided by the trainer prior to, and following, the training session.

Each staff member participated in a semi-structured interview following the training, to capture staff acceptability and practicality. This interview was facilitated online via ZOOM by an experienced researcher (NB) who provided topics and probing questions relating to domains of acceptability of a health care intervention [20], allowing for further exploration of raised contentions. Examples of “acceptability” interview prompts included; How they felt about the intervention (affective attitude); the effort required to undertake the training and use the skills on the ward (burden); the extend to which the training is perceived as likely to achieve its purpose (perceived effectiveness); and confidence that they can perform the new skills and behaviours learnt during training (self-efficacy). The interview was recorded and transcribed verbatim. Along with the semi-structured interview, pre and post RAISE training surveys also explored the staff’s experience of the training program, and the acceptability of incorporating risk assessment (both risk to staff and to the residents) into manual handling tasks when assisting the residents to move around (rated on five levels from ‘very low’ to ‘very high’).

To determine limited efficacy, a researcher observed nursing behaviour while helping the residents to move, pre- and post-training, to report behaviour change which has the potential to reduce nursing injuries and resident falls. These observations reported observable dynamic risk assessment behaviour, which was designed to avoid high-risk assisted movement which could have resulted in a staff injury or a patient fall. That is, if fidelity to the program was achieved. These sessions were conducted for one shift prior to the RAISE training, and then for one shift following the RAISE training. Observations of staff assisting residents to perform mobility activities were compared to the RAISE program competency standards to determine whether the training program resulted in staff behaviour change when assisting residents to perform mobility tasks [11]. The researcher did not intervene or amend the participants’ clinical practice; however, during the observation sessions, the researcher occasionally asked the staff participant about their chosen actions, reasoning processes, and problem-solving approaches during the manual handling task. Observations were by a researcher who was not aware that the staff had participated in RAISE training between the first and second observation.

#### Resident participants

To explore perceptions of residents who received assistance from the staff who participated in the RAISE training program, a short survey was conducted. Qualitative descriptive data were obtained from this convenience sample via a series of questions, framed to review their acceptability of incorporating risk assessment into manual handling when being assisted to move by staff. Example questions included: Do you think that the staff are adequately trained to be able to assist you to move around? Tell me why; The staff ideally want to encourage you to try to do more of the movement for yourself, if you are able. How do you feel about this? Is this acceptable to you? Tell me why; and, The staff have been trained in how to reduce risks. This includes risks to you (such as a fall), and risks to themselves (such as a back injury). Do you think that this is an important part of staff training? Tell me why. The residents were also asked in the survey if they had noted any change to the way staff were helping them to move, over the last few days.

### Outcomes

#### Acceptability (staff and residents)

Staff acceptability of incorporating risk assessment strategies into manual handling approaches was explored through pre- and post-training staff survey; and thematic analysis of the staff semi-structured interview data. Residents’ acceptability of manual handling practices were explored via survey data collected after the RAISE training.

#### Practicality (staff)

Staff perceptions of the practicality of implementing RAISE, including negative impacts or adverse effects, were explored through pre- and post-training staff survey, noting that the participant learning assessed in the surveys aligned to Level 2 of the Kirkpatrick model of evaluation; and thematic analysis of the semi-structured interview data, noting that participant reaction to the training aligned to Level 1 of the Kirkpatrick model of evaluation. Practicality was also measured through demonstrated fidelity to the RAISE program. Fidelity was reported in stages; (i) was there a change in practice; (ii) was the behaviours change according to what had been taught in the RAISE training program; (iii) was this for all movements; and (iv); were all moves performed safely?

#### Limited efficacy testing (staff)

Limited efficacy is based on testing an intermediate outcome, rather than a final outcome [15]. The construct being tested was competency, based on the assumption that demonstrated competency in the RAISE program may result in injuries avoided by the staff, falls avoided for the resident, and movement opportunities being maximised for the resident. To test competency, pre- to post-training changes in staff knowledge and behaviour were captured through: pre- and post-training staff survey (change in knowledge); and observational sessions which focussed on observing staff assisting residents with movement (change in behaviour). Change to clinical practice at the bedside aligned to Level 3 of the Kirkpatrick model of evaluation. Harms and unintended effects were also reported.

### Analysis

Quantitative data on manual handling competency from the staff observational sessions are presented as a number and percentage. Qualitative data from the staff interview and resident surveys are presented descriptively. Two researchers (CG and NB) independently read the transcripts and provided an interpretive description [21], which was mapped to the feasibility domains of acceptability, practicality and limited efficacy. Rigour and trustworthiness of qualitative analysis included the following measures: (a) Themes derived from semi-structured interview data were provided to participating staff to see if they reflect their thoughts and to give them an opportunity to add further ideas (member checking); (b) Interpretive description was completed by two researchers independently; and (c) Collection of data was from multiple sources.

## Results

Data collection occurred in June 2022. There were no harms or unintended effects.

### Participants

#### Staff participants

The study sample included two staff participants; both were female enrolled nurses aged 41–50 years. Another two staff expressed interest in participating but were unable to attend the scheduled afternoon training sessions, so therefore did not participate in the study. Both participating staff members worked full-time, had been employed for over eight years at the long-term care facility, and had worked in healthcare for more than 10 years. Neither staff member had sustained a workplace injury, although one staff participant noted that she experienced intermittent back pain symptoms when performing workplace duties, including resident-assisted movement.

#### Resident participants

The staff identified five residents who met the inclusion criteria. There were no declines to participate, as all five residents were recruited to the project.

### Acceptability and practicality of the RAISE program (staff and residents)

#### Staff participants

Staff indicated in the post-training surveys that they found participation and implementation of the RAISE training program acceptable and practical. They provided positive feedback towards the trainer and the resource materials provided and noted that there was a high likelihood they would implement the learnings from the RAISE program into their working practice. From the semi-structured interview, three themes were identified; two focussed on acceptability and the third on practicality.

#### 1) The RAISE program provided practical guidance

Newly obtained knowledge from the RAISE program improved the staff’s understanding and confidence about manual handling, especially what constituted an acceptable lifting load while employing a risk assessment model of manual handling in their everyday practice.

*‘So, we liked the part where we learnt the seventy-five and twenty-five per cent [rule]. Where the residents do at least seventy-five, then we do no more than the twenty-five’ (Nurse A)*.

The nurses reported that this assisted to form a basis for mapping out risk assessment during mobility tasks with residents. The RAISE program provided guidance about targeted strategies to assist people to move around, which appeared to help to expand the skill set of the staff.

#### 2) The RAISE program enhanced staff empowerment to make decisions

The nurses expressed it was a shift in practice to ask residents to contribute to their transfers, and this had a bearing on the amount of staff-assisted manual handling that needed to be applied.

*‘We were lifting, we were actually lifting their legs, thinking that they were unable to do it. So, in that sense, we now ask them and get them to do a bit more’* (Nurse A).

*‘You have to talk to them, and then give them time to do it. You just have to tell them what you’re doing and then get them to do a bit more’* (Nurse B).

Nurse B reported that she had not experienced any back pain symptoms since RAISE and attributed this to changes in her manual handling techniques, particularly by having the residents contribute more actively to their movements.

#### 3) The need to practise the RAISE program

Throughout the interview, staff reported the need to practise RAISE skills, to consolidate the recently acquired skills, and also embed these into ongoing practice. Staff suggested inclusion of a *buddy system*, a *train the trainer* model and *yearly refresher training* as strategies to support sustainability of the RAISE program in long-term care. The nurses also added that management should provide adequate resources such as dedicated training time and access to equipment, to support the use of RAISE strategies.

#### Resident participants

All residents reported that they require assistance to move from the bed to a chair (n = 5/5; 100%) yet only three required assistance to walk around their room (n = 3/5; 60%). All reported that it is important for staff to be trained in how to assist the residents to move (n = 5/5; 100%), yet only three reported that staff were adequately trained to complete this task (n = 3/5; 60%). Two residents stated that they noticed a change to the way staff assisted their movement following the RAISE training (n = 2/5; 40%). Finally, all residents reported that if staff could spend more time helping them to move around, it would be time well spent (n = 5/5; 100%).

Residents indicated that they found staff using the RAISE training program was acceptable. The five residents considered it was important for staff to be trained how to assist people to mobilise, with one resident additionally highlighting that the staff need to know their situations well to provide person centred care.

*‘They [staff] need to be aware of my individual issues in order to provide the right kind of help’* (Resident 5).

Three of the five residents thought the staff were adequately trained to assist them to move around. When asked how they felt when assisted to move around, most of the residents (n = 4; 80%) displayed an awareness and concern regarding their own movement deficits and indicated that they generally had a good level of confidence due to staff presence. Apprehension about falling was reported by all residents. None of the residents showed awareness of potential risks to staff during manual handling tasks, only the possible risks to themselves when being assisted to move around.

When informed that the staff wanted to encourage them to contribute more to their movement and transfers (as able), the residents’ responses indicated varied acceptability based on their individual abilities and preferences.

*‘Yes, I want to do the most movement that I can’* (Resident 1).

*‘I just want them to do it for me. That is why I live here’* (Resident 5).

All residents reported that clear communication with staff was an important aspect when being supported to move around. The residents considered it would be desirable if the staff could spend more time assisting them to move around, identifying that it would increase their overall activity levels, including ability to access outdoor areas. Throughout many of the survey questions, themes about insufficient staffing and inadequate time availability were evident.

*‘There is not enough staff to spare’ (Resident 3)*.

*‘I would like to walk more. I am dependent on them [staff] having the time.*

*They don’t have the time’* (Resident 1).

Residents generally conveyed that they were satisfied with the care that they received, but consistently discussed that it would be ideal to have greater, and more timely access to the staff members to enable them to be more physically active.

### Limited efficacy testing of the RAISE program (staff)

#### Staff participants

There were 26 observed resident-staff manual handling interactions recorded, with 13 pre-training and 13 post-training (Table 1). After training RAISE skills had improved and were completed 100% of the time, except for completing a physical risk assessment which improved from 46 to 85% (Table 2).

The staff observations also provided evidence of fidelity to the RAISE program. Fidelity was demonstrated through the observed change in practice, where the behaviour change was aligned to what had been taught in the RAISE training program. RAISE skills had improved and were completed, at least in part, for 100% (n = 13) of the observations (Table 2). All observed movements were performed safely and without an adverse event.

**Table 1:** Mobilisation activities that required risk assessment in the long term care setting (2022), n (%)

	Pre-RAISE training (n = 13 episodes of staff assisting resident movement; 23 components of movement)	Post RAISE training (n = 13 episodes of staff assisting resident movement; 23 components of movement)
Transfer components observed* (% is the number of transfer components during the episodes of movement)		
Rolling	5 (38%)	5 (38%)
Moving up / down in bed	1 (8%)	0 (0%)
Sitting up in bed	0 (0%)	2 (15%)
Repositioning in bed	3 (23%)	3 (23%)
Positioning on edge of bed	3 (23%)	2 (15%)
Standing up	2 (15%)	1 (8%)
Stepping and walking	4 (31%)	3 (23%)
Moving back in chair	0 (%)	0 (0%)
Transferring legs into bed	2 (15%)	2 (15%)
Sling hoist	2 (15%)	2 (15%)
Standing machine	1 (8%)	3 (23%)

*Each observation episode may contain several transfer components in sequence.

[Facilitation of step transfers or use of Sara Stedy™ or Patslide™ equipment was not observed to occur during this study, therefore not included as transfer components in the above table]

**Table 2:** Pre and post training audits of RAISE skills in the long term care setting (2022), n (%)

	Pre training (n = 13 episodes of staff assisting resident movement)	Post training (n = 13 episodes of staff assisting resident movement)
**Conducts a physical risk assessment movement**		
Observed	6 (46%)	11 (85%)
Observed with prompts	7 (54%)	2 (15%)
**Verbalises RAISE Concepts**		
**Task Risk Assessment**		
Verbalised	13 (100%)	13 (100%)
Verbalised with prompts	0 (0%)	0 (0%)
**Individual Risk Assessment**		
Verbalised	12 (92%)	13 (100%)
Verbalised with prompts	1 (8%)	0 (0%)
**Load Risk Assessment**		
Verbalised	5 (38%)	13 (100%)
Verbalised with prompts	8 (62%)	0 (0%)
**Environment Risk Assessment**		
Verbalised	12 (92%)	13 (100%)
Verbalised with prompts	1 (8%)	0 (0%)
**Interpretation of Risk Assessment**		
Verbalised	8 (62%)	13 (100%)
Verbalised with prompts	5 (38%)	0 (0%)
**Withdraw from Transfer**		
Verbalised	10 (77%)	13 (100%)
Verbalised with prompts	3 (23%)	0 (0%)
**Demonstrates RAISE Concepts**		
**Safe Staff Positioning**		
Demonstrated	11 (85%)	13 (100%)
Demonstrated with prompts	2 (15%)	0 (0%)
**Appropriate distance from resident when hands-on manual assistance not required (n = 2 episodes)**		
Demonstrated	1 (50%)	2 (100%)
Demonstrated with prompts	1 (50%)	0 (0%)

## Discussion

This study explored the feasibility of implementing RAISE in the long-term care setting. Staff increased their adherence to raise concepts by up to 39% in observation and by up to 62% when asked about reasoning during lifting and handling activities. Staff reported that the RAISE program was acceptable and practical to implement in the long-term care setting. They noted that the RAISE program provided guidance and enhanced staff empowerment to make decisions during assisted resident movement, and there was a need to practise the RAISE program regularly. Residents reported that the RAISE program was acceptable in the long-term care setting and that it was important for staff to be trained on how to assist people to move around. While residents reported they had concerns about themselves falling, they did not acknowledge the potential risks to staff during assisted movement.

This study has several limitations, including a small sample due to the nature of a pilot feasibility study. Chronic staff shortages reduced the number of staff who were able to participate in training and research. Also, we only recruited residents with sufficient level of cognitive functional ability to be able to complete the survey, therefore the results cannot necessarily be generalised to residents with a cognitive impairment. Generalisability is limited as this long-term care facility employs registered and enrolled nurses to meet the minimum staffing to resident ratios, and the personal care attendants are employed as additional support staff above the minimum ratio. This is important to note since in Australia, many long-term care facilities, particularly not-for-profit and private residential aged care providers, have a workforce that predominantly consists of personal care attendants who may have received limited manual handling training due to the brevity of their courses.

While there is a paucity of literature reporting on interventions with demonstrated ability to reduce nurses’ musculoskeletal injuries in the workplace [6, 7], risk assessment has been shown to be vital in determining the resident’s needs [13], indicating the need for a new approach. By embedding comprehensive risk assessment during nurse assisted resident movement, the RAISE program ultimately aims to reduce nursing musculoskeletal injuries; prevent patient falls; and provide opportunities for patients to participate in movement maintenance and rehabilitation. Over the past decade systematic reviews have consistently refuted a causal relationship between nursing staff lower back pain and the daily task of assisting patients with movement [22, 23]. While the RAISE manual handling program does not assume a causal relationship between nursing staff lower back pain and assisting patients with movement, it does assume that through developing competency in RAISE skills, there are avoidable events which occur while assisting patients with movement, that lead to staff injury. For example, lifting a resident when their knees give way during standing, or catching a resident during a fall. The RAISE program focusses on staff behaviour modification, specifically the inclusion of dynamic risk assessment, to identify and avoid the potential risk the adverse event.

We are continuing to further this research program to address current unanswered questions. This ongoing research program will aim to determine if the limited efficacy demonstrated in this pilot study (increase in risk assessment during assisted resident movement) translates to a reduction in staff musculoskeletal injuries through the avoidance of an injury event. It will also aim to determine if this limited efficacy translates to a reduction in resident falls through the avoidance of a high-risk transfer. Finally, this ongoing research program will aim to determine if residents being cared for by RAISE-trained staff participate in more daily movement, aligned to the philosophy of resident participation to the best of their ability. Progression from this pilot study to a future definitive trial will require adaptations based on the staff and resident feedback, such as the practicality of embedded regular RAISE program training into the annual staff education roster.

## Conclusion

This feasibility study identified that the RAISE program was practical and acceptable to staff working in long term care, and that the staff were able to safely adapt their resident manual handling tasks to achieve behaviour change via incorporating a dynamic risk assessment into their daily manual handling tasks. The residents indicated their support of manual handling training programs, and generally highlighted their desire to be able to move around more, but that staffing availability potentially limited the opportunity to do so. This pilot study has justified the inclusion of long-term care settings in future fully powered studies testing the effectiveness and cost-effectiveness of the RAISE patient manual handling program over time and across care staff and residents of different abilities.

## Electronic supplementary material

Below is the link to the electronic supplementary material.

Supplementary Material 1

## Data Availability

Not applicable.

## List of Abbreviations

RAISE: Risk Assessment for moving Individuals SafEly
